# Edentulous disparities among geriatric population according to the sexual difference in South Korea: a nationwide population-based study

**DOI:** 10.1038/s41598-023-35029-3

**Published:** 2023-05-15

**Authors:** Hyang-Ah Park, Soon-Hee Shin, Jae-In Ryu

**Affiliations:** 1 Department of Preventive and Social Dentistry, Graduate School, Kyung Hee University, Seoul, 02447 Korea; 2 Department of Preventive and Social Dentistry, College of Dentistry, Kyung Hee University, Seoul, 02447 Korea

**Keywords:** Dental epidemiology, Geriatrics

## Abstract

The proportion aged 60 years or older in the world's population is expected to double by 2050. In general, they have many complex diseases and poor oral health status. Oral health is one of the important health indicators of elderly people and it is affected by diverse factors, such as socioeconomic status. In this study, sexual difference was considered as an associated factor that is closely related to edentulism. The sexual difference might be more influential within the geriatric population because of lower economic and educational backgrounds at this stage. Edentulism was significantly higher among elderly females than males when combined with the education level. The lower the level of education, the higher the prevalence of edentulism as much as 24 ~ 28 times, especially in females (*P* = 0.002). These findings suggest a more complex relationship between oral health, socioeconomic status, and sexual difference.

## Introduction

According to the United Nation’s World Population Prospects 2019, the geriatric population aged 65 years or older is rapidly increasing and is expected to account for 16% of the world's population by 2050^1^. These population experience problems with vision, hearing, and tooth loss with increasing age^2^ and has at least one restriction in activities of daily living (ADL), such as walking, eating, and washing^3–5^. According to the Health and Retirement Study (HRS) in the United States, almost one-fifth of the elderly with these restrictions answered that their health status was poor^6^. Older females have less access to health care and experience discrimination within the health care system^7^. In particular, the population from lower socioeconomic backgrounds was more likely to report limitations in daily living^8^. This makes the geriatric population more dependent on care. Because many complex diseases already existed, they got less attention for their lower priority, and it makes their oral health condition worse^9^. A decrease in the number of natural teeth makes usual life difficult and health status worse. Intaking of fibrous foods, fruits, and vegetables is decreasing and the risk of gastrointestinal disorders is increasing^10,11^. Tooth loss usually starts from the age of 40 s and rapidly increases when in they were 60 s or 70s^12^. Edentulism is defined as the total loss of all natural teeth and is a significant public health problem worldwide due to its high prevalence and related disorders^13^. According to the US National Health and Nutrition Examination Survey, edentulism was prevalent in 0.7% of the population aged 20–44 years but increased to 20.2% in the geriatric population aged over 65 years^14^. In South Korea, the population with no natural teeth was 0.1% when they were in their 40 s and reached 9.5% at the elderly age of 65 and above^15^.

Tooth loss is affected by various demographic and sociological factors. Studies suggest that the risk factors for edentulism included increasing age^16,17^, poor oral health practices, disability in function^18,19^, low socioeconomic status^20^, and living alone^21^. Elderly people suffer from health and oral health difficulties and the oral health gap to demographic social factors increasingly added and affects their overall life^22^. There are differences by sex in tooth loss as well, more frequently happening in females^23^. Supa et al. mentioned that among males the major factor affecting edentulism was smoking; however, among females, it was lower socioeconomic status^24^. The elderly female showed a fifteen times higher economic dependence than males^25^ and these exacerbate oral health conditions and increase dissatisfaction, depression^26^, or inconvenience in their daily life of them^27^. Sexual differences in health among the elderly have been widely reported^28–30^ but they are not normally considered to be related to disparities, especially in oral health. There is a limited number of studies available to establish the relationship between the diverse variables related to these differences.

This study aimed to understand oral health disparities in the geriatric population, considering sexual difference as a related factor, through analysis of data obtained from the Korean National Health and Nutrition Survey (KNHANES VII, 2016–2018); our findings will aid in the proper planning and implementation of oral health policies.

## Results

### General characteristics of the study population

The total number of participants aged 65 years and older in the 7th National Health and Nutrition Survey (2016–2018) was 3426. Among these elderly participants, 57.6% were female, which was 15% higher than the proportion of than male (Table 1). A great part of the variables, such as educational level, living status, and behavioral factors, showed the proportional difference by sex. Most participants had only elementary school education or none (57.6%). Female had a graduation rate for elementary school, that was 1.5 times the graduation rate of male; however, the number of females graduating high school was only 0.5 times the number of male graduating high school. Twenty-one percent of the participants lived alone; twice as many females lived alone than male. With respect to morbidities, the proportion of the elderly population with hypertension and diabetes was 62.3% and 25.7%, respectively. Female showed a 5% higher rate of hypertension than male, but there was no significant difference in the prevalence of diabetes. The rate of drinking more than once a month was 34.4% and male were a three times higher rate than female. With respect to smoking, 15 times as many males reported a history of smoking than female. Almost 70% of subjects were involved in physical activity on a weekly basis with male reporting a higher likelihood of engaging in physical activity than female. Most people brushed more than twice a day (76.0%); 10% more female brushed more than twice a day than male. Only a quarter of the participants did an annual oral examination; more male received the examination than female.

**Table 1:** Characteristics of the study population of elderly between 2016 to 2018.

Variables	Total		Male		Female		*P-value*
	N	%	n	%	n	%	
Total	3426	100.0	1465	42.4	1961	57.6	
Age							
65–69	1059	31.5	482	33.0	577	30.4	0.483
70–74	916	27.4	387	27.4	529	27.4	
75–79	830	23.7	350	22.5	480	24.6	
≥ 80	621	17.4	246	17.1	375	17.6	
Educational level							
High school and more	834	27.1	557	41.6	277	16.6	< 0.001
Middle school	476	15.3	248	18.6	228	12.8	
Elementary school	1906	57.6	570	39.8	1336	70.6	
Missing value	210		90		120		
Household income quintile							
1st	700	21.1	300	20.9	400	21.3	0.944
2nd	692	19.3	297	19.9	395	18.8	
3rd	697	19.7	296	19.6	401	19.8	
4th	673	20.4	286	20.4	387	20.4	
5th	644	19.5	277	19.2	367	19.8	
Missing value	20		9		11		
Living status							
Alone	853	21.0	201	11.7	652	27.8	< 0.001
With family	2573	79.0	1264	88.3	1309	72.2	
Location of residence							
Province	1852	53.8	762	52.5	1090	54.7	0.203
Metropolitan	1574	46.2	703	47.5	871	45.3	
Hypertension							
Yes	2172	62.3	879	59.4	1293	64.5	0.010
No	1248	37.7	585	40.6	663	35.5	
Missing value	6		1		5		
Diabetes							
Yes	824	25.7	377	26.8	447	24.8	0.288
No	2239	74.3	955	73.2	1284	75.2	
Missing value	363		133		230		
Drinking per month							
Once and more	1144	34.4	813	56.6	331	17.8	< 0.001
Never	2222	65.6	636	43.4	1586	82.2	
Missing value	60		16		44		
Lifetime smoking							
Experienced	1216	35.8	1116	77.0	100	5.1	< 0.001
Never	2146	64.2	330	23.0	1816	94.9	
Missing value	64		19		45		
Aerobic physical activity*							
No	2268	69.0	904	64.1	1364	72.7	< 0.001
Yes	942	31.0	472	35.9	470	27.3	
Missing value	216		89		127		
Tooth brushing per day							
Less than twice	840	24.0	449	30.3	391	19.4	< 0.001
Twice and more	2586	76.0	1016	69.7	1570	80.6	
Oral exam in last year							
No	2567	75.5	1053	72.1	1514	78.1	< 0.001
Yes	795	24.5	393	27.9	402	21.9	
Missing value	64		19		45		

*Medium-intensity physical activity for more than 2 h and 30 min, high-intensity physical activity for more than 1 h and 15 min, or mixed intensity per week (1 min of high-intensity physical activity is equal for 2 min of medium-intensity).

### Prevalence of edentulism in elderly individuals

The proportion of the geriatric population without teeth was 9.5%. This increased with age, with 18.1% of participants aged 80 years being edentulous; the female showed a higher rate of edentulism than the male which was not statistically significant (Table 2). While concerning educational level, the prevalence of edentulism was 11.1% in people who finished elementary school, which was twice as high as that among high school graduates and those with higher education. The gap in the prevalence of edentulism by education level was much higher and statistically different in females compared to males, who did not show statistical significance. The higher the income, the lower the prevalence of edentulism, especially in females; the gap between the first and fifth quartiles was almost double compared to males. The participants who lived alone had a slightly higher prevalence of edentulism than participants who lived with someone else. There was no statistical difference among participants with hypertension in general. However, females with this metabolic disease had a higher prevalence of edentulism. The participants who did not have a history of drinking monthly had a higher prevalence of edentulism, but there was no difference according to the sexual difference. The percent of people with no teeth was 11.4% in smokers, which was higher than that in non-smokers, as much as twice in both males and females. In the case of physical activity, the male who were not engaged in physical activity had a higher rate of edentulism. Participants, both male and female, who brushed lesser than twice a day had a five times higher likelihood of edentulism than those who brushed more. The rate of edentulism was almost five times higher in participants who did not receive oral examinations once a year; the gap was bigger in males, as ten times more likely to be edentulous.

**Table 2:** Prevalence of edentulism according to characteristics of the study population of elderly between 2016 to 2018.

Variables	Total				Male				Female			
	N	n	%	*P-value*	N	n	%	*P-value*	N	n	%	*P-value*
Total	3426	328	9.5	0.597	1465	138	9.1		1961	190	9.8	
Age												
65–69	1059	40	3.9	< 0.001	482	23	4.7	< 0.001	577	17	3.2	< 0.001
70–74	916	64	7.4		387	36	8.8		529	28	6.3	
75–79	830	110	13.1		350	43	12.7		480	67	13.3	
≥ 80	621	114	18.1		246	36	13.5		375	78	21.4	
Educational level												
High school and more	834	49	5.1	< 0.001	557	44	7.2	0.126	277	5	1.2	< 0.001
Middle school	476	33	6.8		248	21	8.2		228	12	5.2	
Elementary school	1906	208	11.1		570	64	11.2		1336	144	11.0	
Household income quintile												
1st	700	84	11.1	0.007	300	34	9.4	0.417	400	50	12.3	0.008
2nd	692	77	11.8		297	33	11.7		395	44	11.9	
3rd	697	71	11.0		396	28	9.6		401	43	12.0	
4th	673	51	7.5		286	25	8.7		387	26	6.6	
5th	644	42	6.1		277	18	6.4		367	24	5.9	
Living status												
Alone	853	117	13.9	< 0.001	201	31	16.2	0.001	652	86	13.2	0.009
With family	2573	211	8.3		1264	107	8.2		1309	106	8.4	
Location of residence												
Province	1852	190	10.5	0.111	762	80	10.3	0.110	1090	110	10.7	0.328
Metropolitan	1574	138	8.3		703	58	7.8		871	80	8.7	
High blood pressure												
Yes	2172	212	9.8	0.324	879	75	7.8	0.065	1293	137	11.1	0.004
No	1248	113	8.8		585	63	11		663	50	6.9	
Diabetes												
Yes	824	85	9.9	0.171	377	38	8.9	0.751	447	47	10.6	0.153
No	2239	183	8.0		955	82	8.3		1294	101	7.7	
Drinking per month												
Once and more	1144	89	7.7	0.044	813	64	8.0	0.106	331	25	7.0	0.124
Never	2222	224	10.0		636	73	10.7		1586	151	9.7	
Lifetime smoking												
Experienced	1216	139	11.4	0.008	1116	123	10.7	< 0.001	100	16	19.5	0.008
Never	2146	174	8.0		330	14	4.1		1816	160	8.7	
Aerobic physical activity*												
No	2268	224	9.8	0.105	904	89	10.4	0.011	1364	135	9.4	0.479
Yes	942	68	6.8		472	39	6.1		470	29	7.4	
Tooth brushing per day												
Less than twice	840	181	21.7	< 0.001	449	85	19.2	< 0.001	391	96	24.6	< 0.001
Twice and more	2586	147	5.6		1016	53	4.7		1570	64	6.2	
Oral exam last year												
No	2567	295	11.6	< 0.001	1053	130	12.2	< 0.001	1514	165	11.1	< 0.001
Yes	795	18	2.1		393	7	1.3		402	11	2.7	

*Medium-intensity physical activity for more than 2 h and 30 min, high-intensity physical activity for more than 1 h and 15 min, or mixed intensity per week (1 min of high-intensity physical activity is equal for 2 min of medium-intensity).

### Logistic regressions by risk factors associated with edentulism in elderly individuals

The logistic regression analysis for the factors affecting the prevalence of edentulism among elderly individuals is presented in Table 3. Age, educational level, income, living alone, and lifetime smoking were significantly associated with edentulism among elderly individuals. Participants who brushed fewer than twice a day were five times more likely to be edentulous than participants who did, and participants who did not receive yearly oral examinations were six times more likely to be edentulous than participants who did. After adjustment for all risk factors, the geriatric population who did not receive yearly oral examinations showed a lower odds ratio: almost four times that before. The coefficient of determination of NagelKerke R2 in this model was 0.185. There were different patterns according to the sexual difference in the prevalence of edentulism and educational level in Tables 1 and 2. In the third model considering the interaction effect of educational level to edentulism depending on the sexual difference, the odds ratio of edentulism decreased for females with the opposite results of those from the second model. Compared to the group of elderly female participants who graduated high school, people who finished middle school showed 29 times higher risk of being edentulous, and people who graduated elementary school were at a 24-fold higher risk of being edentulous. The lower the level of education, the higher the prevalence of edentulism, especially in females. The coefficient of determination of NagelKerke R2 in the final model was 0.198 and the p-value was 0.006 for the test of model effects of interaction between the sexual difference and educational level.

**Table 3:** Odds ratio (OR) and 95% confidence interval (CI) estimated from logistic regression model for edentulism of elderly between 2016 to 2018.

Variables	No-interaction						Interaction (sex*educational level)		
	Unadjusted			Model 1			Model 2		
	OR	95% CI	*P-value*	OR	95% CI	*P-value*	OR	95% CI	*P-value*
Sex (= male)									
Female	1.079	0.813–1.433	0.597	1.507	0.740–3.071	0.258	0.074	0.010–0.576	0.013
Age (= 65–69)									
70–74	1.977	1.211–3.229	0.007	2.049	1.225–3.428	0.006	2.130	1.269–3.576	0.004
75–79	3.745	2.300–5.393	< 0.001	3.571	2.053–6.210	< 0.001	3.486	1.999–6.080	< 0.001
≥ 80	5.507	3.619–8.378	< 0.001	3.382	1.967–5.816	< 0.001	3.325	1.935–5.713	< 0.001
Educational level (= high school and more)									
Middle school	1.364	0.817–2.276	0.234	1.440	0.809–2.563	0.215	0.908	0.465–1.773	0.777
Elementary school	2.337	1.592–3.430	< 0.001	1.453	0.901–2.342	0.125	0.936	0.524–1.673	0.824
Household income (= 5th)									
4th	1.249	0.766–2.037	0.371	1.168	0.660–2.065	0.594	1.121	0.634–1.981	0.694
3rd	1.914	1.148–3.191	0.013	1.247	0.631–2.462	0.525	1.198	0.613–2.342	0.598
2nd	2.074	1.310–3.283	0.002	1.036	0.561–1.914	0.909	1.015	0.554–1.859	0.961
1st	1.935	1.222–3.064	0.005	0.880	0.437–1.711	0.719	0.852	0.428–1.697	0.649
Living status (= with family)									
Alone	1.791	1.332–2.407	< 0.001	1.502	0.936–2.409	0.092	1.508	0.936–2.432	0.091
Location of residence (= metropolitan)									
Province	1.304	0.940–1.808	0.111	1.202	0.825–1.751	0.336	1.227	0.841–1.790	0.288
High blood pressure (= no)									
Yes	1.133	0.884–1.453	0.324	0.858	0.638–1.153	0.310	0.875	0.651–1.175	0.373
Diabetes (= no)									
Yes	1.259	0.905–1.751	0.172	1.008	0.705–1.441	0.966	1.006	0.706–1.433	0.976
Drinking per month (= never)									
Once and more	0.749	0.565–0.992	0.749	0.854	0.526–1.081	0.124	0.748	0.523–1.071	0.113
Lifetime smoking (= Never)									
Experienced	1.481	1.139–1.927	0.003	2.403	1.295–4.460	0.006	2.346	1.268–4.341	0.007
Aerobic physical activity* (= yes)									
No	1.483	0.919–2.393	0.107	0.922	0.563–1.509	0.746	0.920	0.562–1.506	0.739
Tooth brushing per day (= Twice and more)									
Less than twice	4.672	3.501–6.235	< 0.001	3.471	2.502–4.815	< 0.001	3.548	2.559–4.919	< 0.001
Oral exam in last year (= Yes)									
No	6.222	3.657–10.586	< 0.001	3.807	2.071–6.995	< 0.001	3.743	2.040–6.868	< 0.001
Sex*Educational level (= female*high school and more)									
Female*middle school							28.877	3.488–238.080	0.002
Female*elementary school							24.422	3.272–182.275	0.002
NagelKerke R2				0.185			0.198		

*Model 1: fully adjusted for sex, age group, educational level, household income quintile, living status with family, location of residence, existence of high blood pressure or diabetes, drinking, smoking, physical activity, frequency of tooth brushing, and oral exam experience in last year, Model 2: Model 1 plus interaction between sex and educational level.

## Discussion

The proportion of the world's population aged 60 years or older is expected to double by 2050. As a result, population aging is recognized as an important problem worldwide^31^. Oral health is an essential health indicator among elderly people^9,10^. A gap is reported in the oral health of geriatrics for various factors including socioeconomic and behavioral factors. In this study, sexual difference was considered the main factor that affected the prevalence of edentulism among those aged 65 years or above.

There was no difference in the prevalence of edentulism according to the sexual difference until the interaction between the sexual difference and education level was considered, which was significant. Edentulism among elderly females was significantly higher than that among males when categorized by education level. The prevalence of edentulism among middle school or elementary school graduates and participants with lower education levels was 24–28 times that of high school graduates or participants with higher education in an elderly female. The prevalence of edentulism among elderly individuals in South Korea was 9.5%, which was lower than the prevalence of 12.9% observed in the United States^32^, but higher than the prevalence of 8.1% observed in Japan^33^. The proportion of elderly individuals is expected to reach 46.5% in 2067^34^, of which the proportion of female elderly individuals is expected to be about 55%, higher than that of male elderly individuals in Korea. Sexual difference may be an important factor associated with edentulism among the geriatric population, especially for economic and educational backgrounds. Some studies have reported that the lower the education or income, the higher the prevalence of edentulism^35–38^. This population is also reported to have lower subjective oral health, which might affect care for oral health^39^. Most of them showed a lower tendency to visit dental clinics^40^, which might have worsened oral health conditions. There was a significant difference in edentulism by socioeconomic level in an elderly female. This is the reason that the interaction between sex differences and socioeconomic factors is assumed to exist. There might be a more complex relationship between oral health, socioeconomic status, and sexual difference. Among the geriatric Korean population, a female had a significantly less chance than a male of being educated. They also lived longer than males and many of them lived alone^41^. Elderly females were found to be more sensitive to socioeconomic factors as well. The gap in economic poverty appeared in poorer elderly females, which worsened oral health^42^. Paola et al. reported that elderly females did more preventive oral care than males, but complex mechanisms working in oral health make necessary careful management^43^. The oral health gap should be considered not just by the sexual difference itself but also by the contextual mechanism around it.

Regular tooth brushing twice a day and yearly oral examination was found to have effects on edentulism. Healthy behaviors were reported as closely related to oral health^44^. The prevalence of edentulism was higher in participants with a history of smoking, brushing fewer than twice a day, and not taking yearly oral examinations^45,46^. These oral health behaviors showed different patterns depending on the sexual difference, and females are known to visit the dentist more frequently, perform better oral care^47^, and have better knowledge, attitude, and behavior about oral health than males^48,49^. The elderly female showed about a 10% higher rate of brushing their teeth twice or more a day than males in this study. Likewise, the sexual difference in elderly individuals had significant associations with different oral health behavior factors, such as physical activity for males and hypertension for females. The type of residence also showed a significant association. Elderly individuals living alone were at an almost two times higher risk of edentulism. Kim et al. reported that elderly people who lived alone had a higher possibility of needing dentures and experiencing difficulty in mastication than geriatric people living with their families^50^. Elderly males living without families showed a higher level of edentulism. Elderly individuals living alone might have fewer opportunities to acquire and exchange information about oral health and more difficulties in practicing healthy oral behavior^51^.

There are several limitations of this study. First, social relationships and support might influence physical, mental, and oral health and behavior, especially in geriatric people^52,53^. The data used in this study did not include social capital variables such as the number of friends and meetings. It is necessary to understand the social relationship between elderly individuals with oral health in depth in the future. Second, it is difficult to identify the causal relationship because the data was a cross-sectional study. The present education or income levels were surveyed but the past socioeconomic status or oral health behavior of the participant could not be included in this survey. The causal relationship from previous conditions would be important in the study of the geriatric population. Third, this study focused on edentulism disparities among the geriatric population according to sexual differences and educational levels. There might have been other confounding factors with sex, such as hypertension and aerobic physical activity. When they were added to interaction terms analysis and few minor numeric changes of coefficients in the model existed and the p values lay on the borderline of statistical significance. More to the point, it was not clear whether sex was a confounding factor with these variables in the contextual framework. Therefore, only educational level was included as an interaction term in the final model. Future studies must design a longitudinal data set such as a cohort framework to identify the influential relationship after identifying the related factors including diverse confounders. Even with these limitations, this study established the fundamentally important health characteristics of an aging society according to the sexual difference with oral health status using nationally representative data.

## Conclusion

This study revealed the association between sex differences and socioeconomic factors related to the oral health of elderly individuals. In edentulism in elderly individuals, there was no difference according to the sexual difference, initially. After considering the interaction between sex difference and education, an elderly female had a lower possibility of being edentulous than a male. The prevalence of edentulism in the elderly female who graduated middle school or elementary school was 24 ~ 28 times that of high school graduates. The sexual difference in the geriatric population had significant associations with different oral health behavior factors, such as physical activity for males and hypertension for females. In conclusion, in the plan for oral health improvement in elderly individuals, the sexual difference must be identified, such as socioeconomic factors for females and health behavior factors for males.

## Methods

### Study subjects

This study analyzed oral health conditions by the sexual difference in the geriatric population aged 65 years or older using the Korean National Health and Nutrition Survey (KNHANES VII, 2016–2018) data. The sampling frame was layered based on the size of the area (cities, provinces, and districts) and housing types (general housing, apartments). The ratio of residential area and educational background of household owners was used as the intrinsic stratification criteria. Finally, 576 districts were surveyed over 3 years, with 10,611 households participating in the study. A cohort of 24,269 participants was recruited with a response rate of 76.6%. Among them, 3426 people were elderly, which was 21.0% of all subjects.

### Study variable

The general characteristics of the study sample were sex, age, education or income level, household type, region, comorbidities such as hypertension or diabetes, health-related behavior such as drinking, smoking, aerobic physical activity, brushing teeth, or visiting a dental clinic for oral examination per year. The independent variables are classified as shown in Table 1. The type of household was categorized by the number of family members—one as living alone and two or more as a family living together. A person involved in as much as medium-intensity physical activity for 2 h and 30 min, high-intensity physical activity for 1 h and 15 min, or medium and high-intensity physical activity per week (1-min-high intensity for 2 min) was defined as properly engaging in physical activity. Edentulism was defined as a person who had none of the natural teeth including third molars, calculated based on the result of the oral examination. A complex sample logistic regression could not check the collinearity of the variables in the options of the statistical package. Therefore, we applied the collinearity option in the general logistic regression with study variables. Variance inflation factors (VIF) were used to assess multicollinearity among the socioeconomic variables. VIF > 10 indicated the presence of multicollinearity^54^. However, no indicators of multicollinearity were identified as all the VIFs were under 5.

### Statistical analysis

A complex sample analysis was used because the KNHANES was a two-stage stratified cluster sampling. It was conducted by generating an integrated weight based on the prepared analysis plan file. The plan file adapted analysis weight with “oral examination weight”, design of the strata with “variance estimation” and “group aged 65 years or above, and cluster as primary sampling unit with the district. The KNHANES recommended analyzing the data by reflecting this sample design (stratum, cluster, weight). Especially if only a part of the data in a complex sample is analyzed, the standard error of the estimate might be biased due to missing data information. The group variable as the elderly “aged 65 years or above” was created and adapted for a subgroup analysis^55,56^. Complex sample frequency and chi-square tests were conducted to find out the difference in the prevalence of edentulism according to socioeconomic factors, chronic diseases, and health-related behaviors. A complex sample logistical regressions were performed with an unadjusted model with univariate variables, a fully adjusted model including all variables (Model 1), and an advanced model with interaction by the sexual difference and education (Model 2). An interaction analysis was conducted to confirm the relationship between education level and edentulism depending on the sexual difference. After including the interaction terms of sex and education variables in the complex logistic regression model, the significance of each interaction term confirmed whether edentulism appeared differently by education level according to the sexual difference. All analyses used SPSS (Statistical Packages for Social Science 26.0. SPSS Inc., USA) and statistical significance was set to α = 0.05.

### Ethical approval and informed consent

This study used the dataset obtained from the KNHANES VII, 2016 to 2018. All KNHANES were conducted with participants’ informed consent after approval by the Research Ethics Review Committee of the Korea Disease Control and Prevention Agency (KDCA) (IRB No. 2018–03-P-A for the KNHANES VII). This analytical study was approved again by the institutional review board (IRB) of Kyung Hee University (IRB No. KHSIRB-21–337(EA)) as exemption of the review because this retrospective analysis included the dataset of national surveillance and did not contain personally identifiable information. All methods were carried out following the KNHANES analytic guidelines and regulations.

## Data Availability

The data that supports the findings of this study are available from the Korean Disease Control and Prevention Agency (KDCA), but restrictions apply to the availability of data, which was used with permission for the current study and therefore not publicly available. Data is however available upon reasonable request and with permission of KDCA.
